# Assessment of the knowledge and awareness of toxoplasmosis among doctors and nurses in Casablanca, Morocco: a cross-sectional study

**DOI:** 10.11604/pamj.2025.50.30.45541

**Published:** 2025-01-20

**Authors:** Majda Laboudi, Ait Hamou Sanaa

**Affiliations:** 1 Department of Parasitology, National Institute of Hygiene, Rabat, Morocco,; 2 Laboratory of Ecology and Environment, Faculty of Sciences Ben M'Sik, Hassan II University, Casablanca, Morocco

**Keywords:** Toxoplasmosis, doctors, nurses, knowledge, Casablanca, Morocco

## Abstract

**Introduction:**

toxoplasmosis is a zoonotic infectious disease caused by Toxoplasma gondii. Medical health professional specially doctors and nurses must have the best skills, knowledge and practices regarding toxoplasmosis to improve maternal and newborn health effectiveness. The aim of this current survey was to assess the knowledge of toxoplasmosis among doctors and nurses, working in selected health facilities of different parts of Casablanca prefecture in Morocco.

**Methods:**

a cross-sectional study was conducted. The data were collected from consenting doctors and nurses through a self-administered structured questionnaire. The questionnaire included the sociodemographic and knowledge regarding the general information, diagnosis, clinical and prevention of toxoplasmosis. Data were analyzed using descriptive statistics and association between variables explored with Chi-square test at P < 0.05.

**Results:**

one hundred and twenty-six health professionals participated in the study with mean age was 40.50 ±10.06 years. Of these, 83.3% were females while 16.7% were male. Fifty two (41.3%) of the respondents were doctors while 74 (58.7%) of the respondents were nurses. More than half of respondents (57.1%) had a moderate knowledge score about toxoplasmosis with doctors had better knowledge of toxoplasmosis than did nurses. Nineteen-five percent (95%) of respondents were aware of the definition of toxoplasmosis and more than 80% knew about the risk factors of this zoonosis. The majority of doctors and nurses (more than 90%) recognized the measures to follow to avoid the transmission of toxoplasmosis for pregnant women such as no contact with cats, eating cooked meat, washing their hands often and washing fruits and vegetable products before eating. However, the most of respondents were unaware of avidity test (91.7%).

**Conclusion:**

we concluded that the knowledge about various aspects of toxoplasmosis was average among medical health professionals in Casablanca. Therefore, educational intervention for nurses and doctors contributed to improving knowledge about the disease to reduce exposure the pregnant women to some risk factors of disease during pregnancy.

## Introduction

Toxoplasmosis is one of neglected zoonotic parasitic diseases, it's caused by a protozoan, opportunistic parasite, *Toxoplasma gondii* (*T. gondii*) [1]. It is acquired through the consumption of undercooked meat containing tissue cysts or ingestion of oocysts excreted by cats or contaminated soil or water [2]. Also by organ transplantation, blood transfusion or congenital transmission [3,4]. All warm-blooded animals are intermediate hosts for *T. gondii*; however, the only definitive hosts of *T. gondii* are felids [5].

Toxoplasmosis is a benign disease in immunocompetent people, but it is, however, a potentially serious infection in pregnant women, due to the risk of congenital toxoplasmosis with neurological and ophthalmological damage in the event of fetal transmission [4,6]. The overall annual incidence of congenital toxoplasmosis worldwide has been estimated at 190,100 cases. The highest incidence rates were observed in South America, some Middle Eastern countries, and other low-income countries [7]. In general, there are three possible levels to prevent toxoplasmosis in pregnant women: primary, secondary and tertiary. Primary prevention is the most effective and recommended measure against congenital toxoplasmosis [8]. This type of prevention is a priority in most countries and supported by the Centers for Disease Control and Prevention (CDC), the latter recommends the adoption of individual measures for primary prevention of toxoplasmosis during pregnancy, with a preventive behavior oriented towards food hygiene [9]. Also, among the main measures of this prevention, is to identify the risk factors of toxoplasmosis during pregnancy and to provide advice to pregnant women who present a negative serology for toxoplasmosis during the first prenatal consultation [10]. This approach cannot eliminate the risk of a woman being infected with *T. gondii*, but significantly reduces the rate of seroconversion (infection) during pregnancy [8].

In 2009, the International Federation of Gynecology and Obstetrics (FIGO) worked toward the overarching goal of reducing maternal and newborn mortality and morbidity by implementing the project “Improving maternal and newborn health in low-resource countries through strengthening the role of obstetric and gynecological national associations” [11]. The FIGO has issued recommendations for perinatal health professionals and acknowledges their key role in the primary prevention of toxoplasmosis in pregnant women [11]. Indeed, Health professionals are responsible for conducting educational activities for women with high-risk pregnancies during prenatal consultations. The paramount importance of the healthcare professionals' role in all pregnancy processes is mentioned, including detection, and guidance during a pregnancy. Therefore, medical health professionals (MHPs) need to have sufficient knowledge, experience, and skills to promote maternal and newborn health effectively. In Morocco, the prevalence of infection among the pregnant population is 43% with 48.8% of pregnant women presenting themselves for serological screening for toxoplasmosis for the first time in the second trimester of pregnancy [12]. This situation has aroused great interest and questions us about the state of knowledge of this disease among the MPHs regarding toxoplasmosis. Therefore, the present study aimed to assess the awareness and practices regarding toxoplasmosis among MHPs providing antenatal care services at health facilities in Casablanca, Morocco.

## Methods

### Study design

A cross-sectional study was undertaken in 34 health centers in Casablanca prefecture in Morocco between January and May 2018 in the urban areas of Casablanca. Thus, the health centers were randomly chosen from the list in web portal of the Moroccan Ministry of Health.

### Study setting and population

**Figure 1:**
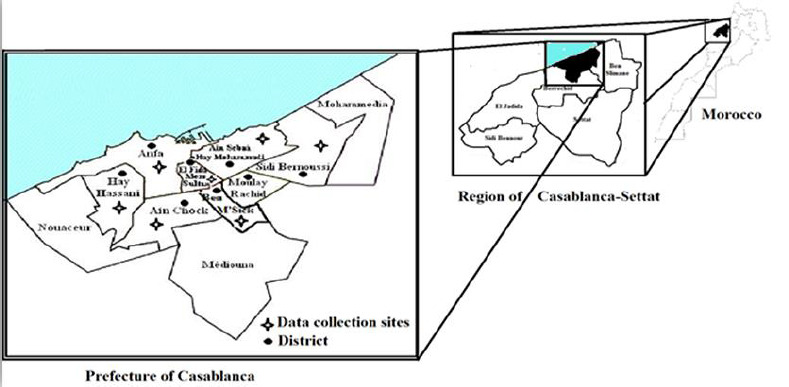
map of Morocco, region of Casablanca-Settat, prefecture of Casablanca with urban health centers selected

The prefecture of Casablanca is an exclusively urban subdivision of the Moroccan region of Casablanca-Settat. It is located on the Atlantic coast of the country; It is subdivided into 8 prefectures of districts. The prefecture of Casablanca comprises two urban communes (or municipalities): the urban municipality of Casablanca, which includes eight district prefectures: the prefecture of Aïn Chock, the prefecture of Aïn Sebaâ-Hay Mohammadi, Al Fida-Mers Sultan prefecture, the prefecture of Ben M'sick, the prefecture of Casablanca-Anfa, the prefecture of Hay Hassani, the prefecture of Moulay Rachid and the prefecture of Sidi Bernoussi. The population of the municipality of Casablanca increased, from 1994 to 2018, from 2,717,125 to 4,500,000 inhabitants area (Figure 1).

The study population was medical health professionals such as the doctors (general practitioners, pediatricians, and gynecologist doctors), and nurses (versatile nurses). The inclusion criteria were doctors and nurses who agreed to be involved in this study and completed the questionnaire and practicing in urban public health centers selected in the prefecture of Casablanca in Morocco. The participants who did not meet the inclusion criteria were excluded from the study.

### Variables

The knowledge of toxoplasmosis was the outcome variable which was defined in the two groups of health professionals: doctors and nurses. Independent variables included socio-demographic characteristics (age, gender, education level, length of service years in public center health) and responses for knowledge regarding toxoplasmosis (epidemiology, diagnosis, clinical and prevention).

### Data resource and measurement

#### Data collection tool : questionnaire

The questionnaire was composed of two sections: the first concerned demographic characteristics of the participants (gender, age, the year of service and education level) and the second, knowledge about toxoplasmosis (epidemiology, clinical manifestations, diagnosis, treatment and prevention). The questionnaire was designed in French language and was anonymous and was filed in the workplaces of the participants. The objective of the study was explained to the doctors and nurses before they were asked to complete the questionnaire.

Pretesting of the questionnaire was undertaken on 10 health professionals attending one of the two chosen public centers in prefecture in order to check for the feasibility and understandability of the questionnaire. These samples were not included in the sample studied.

### Sample size

The sample size was estimated using the formula recommended by the World Health Organization [13]. The following criteria were established: the adequate knowledge rate at 50%, the confidence level at 95%, and the margin of error at 5%. The total number involved in this study was 126 according to the following criteria: health professionals who accepted to participate in this study and completed the questionnaire.

The recruitment of our study population based on convenience. All physicians and nurses who were present on the day of data collection were invited to participate in the survey.

### Scoring of knowledge

Scoring system of participants' knowledge was done as followed: three response scales were used, in which doctors and nurses were required to give 'correct', 'incorrect', or 'don't know' answer to all questions. The response of each knowledge question was scored as score 1 for correct response and score 0 for incorrect or don't know response. The total score of each participant was converted to a percentage. More than 75% of score were scored as a high level of knowledge, those with 50-75% were treated as having an average level of knowledge and those with less than 50% were considered as a low level of knowledge.

### Data analysis

Statistical analysis was computed using EPINFO software (Version 3.5.4, 2012). The descriptive statistical analysis of the demographic variable characteristics of participants (such as gender, age, length of service years in public center health and education level) was performed and results were presented in frequencies (n) and percentages (%). A bivariate analysis was undertaken to assess the relationship between the doctors and nurses with their socio-demographic characters. Pearson Chi-Square test was conducted to ascertain the statistical significance. And it was also used to assess the possible associations, between the knowledge of toxoplasmosis items and the type of profession of respondents. For tables with lower counts (less than five), Fisher's exact test was used. Statistical significance was tested at 5% significant level or 95% confidence interval. All significance values less than or equal to 0.05 were considered to be statistically significant.

### Ethics approval and consent to participate

The Research Ethics Committee of the Faculty of Medicine and Pharmacy, Casablanca, Morocco, approved the protocol of this study (Number: 20/17). Moreover, permission was obtained from regional director of permission manager for their help during the performance of a study. Written informed consent was obtained from HPs before data and sample collection after explaining to them the aim and procedures of the study.

## Results

### Socio-demographics characteristics of participants

Approximately 195 questionnaires were distributed to doctors and nurses participants during the study period. The questionnaire was filled completely by 126 participants with a 64.2% response rate. A total of 69 questionnaires was excluded because either the participants were not doctors (n=2) or not nurses (n=5) or incomplete data (n=24). In the present study, 126 individuals agreed to participate in this study and filled completely the questionnaire were interviewed from 34 facilities, health of the Casablanca prefecture including 58.7% nurses and 41.3% doctors. Of these, 83.3% were females while 16.7% were male. The mean age was 40.50 ±10.06 years. The majority of whom were aged more than 45 years, 36.5% while 8.7% were in the 31-35 years age group. The education level characteristics of the studied population indicated that most of the respondents had a baccalaureate add three years, 56.3% (Table 1). Regarding the year of service of the respondents, the average of professional experience was 14.42 ± 8.59 years. 42.9% served more than 15 years, 19.8% served 11-15 years, and lastly 37.3% served for less than ten years.

**Table 1:** professional and sociodemographic characteristics of participants

	N	%
**Age: Mean:** 40.51 ±10.06		
<30	30	23.8%
31-35	11	8.7%
36-45	39	31.0%
46-60	46	36.5%
**Gender**		
Woman	105	83.3%
Man	21	16.7%
**Profession**		
Nurses	74	58.7%
Doctors	52	41.3%
**Education level: Time from graduation after baccalaureate (years)**		
≤ 3	71	56.3%
4 - 7	32	25.4%
> 7	23	18.3%
**Length of service years in public center health (years) Mean:** 14.42 ± 8.59		
10	47	37.3%
11-15	25	19,8%
>15	54	42.9%

Table 2 shows relationship between nurses and doctors with their socio-demographic characters. There is no statistically significant difference (p>0.05) between profession and gender. As regards age, educational level and length of service years in public center health, there is a statistically significant difference (p<0.05) (Table 2).

**Table 2:** relationship between doctors and nurses with sociodemographic characters in health facilities in Casablanca

		Nurses N= 74 (%)	Doctors N=52 (%)	Total N= 126 (%)	CI 95%		X^2^	p
**Gender**								
	Women	65 (61.9)	40 (38.1)	105 (83.3)	75.7%	89.4%	2.61	0.105
	Man	9 (42.9)	12 (57.1)	21 (16.7)	10.6%	24.3%		
**Age ( years) Mean:** 40.51 ± 10.06								
	<30	25 (83.3)	5 (16.7)	30 (23.8)	16.7%	32.2%	20.92	0.000*
	31-35	9 (81.8)	2 (18.2)	11 (8.7)	4.4%	15.1%		
	36-45	24 (61.5)	15 (38.5)	39 (31)	23.0%	39.8%		
	>45	16 (34.8)	30 (65.2)	46 (36.5)	28.1%	45.6%		
**Length of service years in public center health (years) Mean** 14.42 ± 8.59								
	≤ 10	34 (72.3	13 (27.7	47 (37.3)	28.9%	46.4%	6.16	0.045*
	11-15	14 (56.0)	11 (44.0)	25 (19.8	13.3%	27.9%		
	>15	26 (48.1)	28 (51.9)	54 (42.9)	34.1%	52.0%		
**Education level: time from graduation after baccalaureate ( years)**								
	≤ 3	71 (100)	0 (0.0)	71 (56.3)	47.2%	65.2%	114.78	0.000*
	4-7	3 (9.4)	29 (90.6)	32 (25.4)	18.1%	33.9%		
	> 7	0 ((0.0)	23 (100)	23 (18.3)	11.9%	26.1%		

### Level of knowledge among the doctors and nurses regarding toxoplasmosis

**Figure 2:**
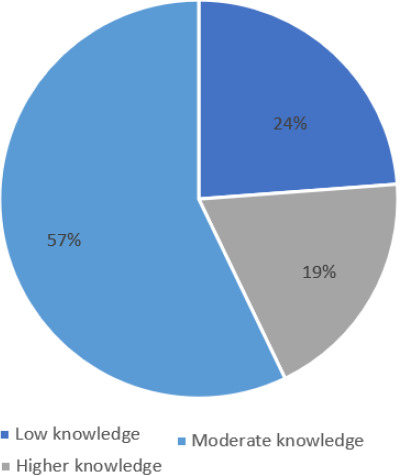
level of knowledge among the nurses and doctors regarding toxoplasmosis

More than half (57.1 %) of doctors and nurses participants had a moderate knowledge score and less than half (23.8%) of the sample had low knowledge about toxoplasmosis while only 19 % had a good knowledge about toxoplasmosis (Figure 2). Regarding the relationship between the quality of professional and the level knowledge, the results shows that the doctors had higher knowledge than the nurses. This result is statistically significant (p<0.05).

### Knowledge of toxoplasmosis risk factors

The majority of doctors and nurses knew about the definition of toxoplasmosis due to a parasite of *T. gondii* (95.2%) and more than (87.3%) knew about the definitive host of toxoplasmosis (Table 3). Regarding the risk factors, 85.7 % of participants correctly considered that the direct contact with cat to be the risk factor for toxoplasmosis transmission. Many of the respondents (92.9%) correctly considered the consumption of raw or undercooked meat as the route of transmission of toxoplasmosis, while 65.9% of participants related transmission of toxoplasmosis to untreated water (Table 3).

**Table 3:** epidemiology knowledge among doctors and nurses regarding toxoplasmosis

	Nurses		Doctors		Total N= 126	%	X^2^	p
	N = 74	(%)	N = 52	(%)				
**Epidemiology**								
**Do you know the agent of the toxoplasmosis is T. gondii?**								
Yes	69	93.2	51	98	120	95.2	1.9172	0.3834
No	5	6.7	1	1.9	6	4.7		
**Do you know the definitive host of toxoplasmosis?**								
Yes	65	87.8	45	86.5	110	87.3	0.0465	0.8292
No	9	12.2	7	13.5	16	12.7		
**Risk factors: Infection with *T. gondii* can be acquired by**								
**Direct contact with cat**								
Yes	62	83.8	46	88.5	108	85.7	0.9744	0.6143
No	4	5.4	3	5,8	7	5.6		
Don't know	8	10.8	3	5,8	11	8.7		
**Fruits and vegetables having contact with cat stool**								
Yes	69	93.2	49	94.2	118	93.7	4.6916	0.0958
No	1	1.4	3	5.8	4	3.2		
Don't know	4	5.4	0	0.0	4	3.2		
**Drinking untreated water**								
Yes	44	59.5	39	75.0	83	65.9	5.3816	0.0678
No	25	33.8	8	15.4	33	26.2		
Don't know	5	6.8	5	9.6	10	7.9		
**Eating undercooked meat**								
Yes	67	90.5	50	96.2	117	92.9	3.7429	0.1539
No	2	2.7	2	3.8	4	3.2		
Don't know	5	6.8	0	0.0	5	4.0		

### Knowledge about toxoplasmosis diagnostic treatment clinic and prevention

Respondents generally had a good knowledge of the appropriate test for diagnosis of toxoplasmosis. However, the results indicated that allow knowledge of the respondents related to avidity test (8.3%). In contrast, the survey revealed that most of the people were aware that the principal diagnosis serologic is the research of IgG and IgM antibodies of *T. gondii* (Table 4). Concerning the diagnosis of congenital toxoplasmosis, only 27.8% know the diagnosis is based on amniotic by Polymerase Chain Reaction (PCR). On the other side, only 27.8% of MHPs don’t know about it the treatment in case of seroconverssion to prevent the transmission of the parasite to the fetus. Half of MPHs (51.6%) know that the prophylaxis of toxoplasmosis must be recommended to seronegative pregnant women. Besides, 65.9 % of MHPs correctly believed that vaccination does not exist in controlling human toxoplasmosis. The results were insignificantly different between doctors and nurses. Most of MHPs knew about the definitive host of disease definition of toxoplasmosis due to a parasite of *T. gondii* (95.2%). Even though the majority knew (74.6 %) of the definition of seroconversion only 31.7% responded correctly about the definition of congenital toxoplasmosis Additionally, approximately 67.7% correctly responded that toxoplasmosis is opportunist disease in immunocompromised patients. The study found that the majority of participants (82.5%) were aware of that in the majority cases of toxoplasmosis in immunocompetent person are asymptomatic (Table 4). Regarding prevention knowledge, more than 90% of MHPs recognized the measures to follow to avoid the transmission of toxoplasmosis for pregnant women such as no contact with cats, eating cooked meat, washing their hands often and washing fruits and vegetable products before eating (Table 5).

**Table 4:** diagnosis, clinical treatment and prevention knowledge among doctors and nurses regarding toxoplasmosis

	Nurses									Doctors		Total N=126	%	X^2^	p
	N= 74								(%)	N=52	(%)				
**Diagnosis**															
**Do you know that the antenatal screening for T. gondii infection is based on anti-toxoplasma-specific IgG and IgM detection?**															
Yes	61								82.4	45	86.5	106	84.1	3.24	0.198
No	4								5.4	5	9.6	9	7.1		
Don’t know	9								12.2	2	3.8	11	8.7		
**Do you know the anti-toxoplasma IgG avidity test?**															
Yes	5								7.0	5	0.0	10	8.3	0.33	0.5606
No	66								93.0	45	90.0	111	91.7		
**Do you know that the diagnosis of congenital toxoplasmosis is based on amniotic by PCR?**															
Yes	10								14.9	22	45.8	32	27.8	17.14	0.0002*
No	4								6.0	6	12.5	10	8.7		
Don’t know	53								79.1	20	41.7	730	63.5		
**Do you know the treatment in case of seroconversion to prevent the transmission of the parasite to the fetus?**															
Yes	8								10.8	28	53.8	36	28.6	27.71	0.0000*
No	66								89.2	24	46.2	90	71.4		
**Do you know that the prophylaxis of toxoplasmosis must be recommended among seronegative pregnant women?**															
Yes	33								44.6	32	61.5	65	51.6	6.29	0.0431
No	13								17.6	11	21.2	24	19.0		
Don’t know	28								37.8	9	17.3	37	29.4		
**Does the human vaccine of toxoplasmosis exist**															
Yes	6								8.1	4	7.7	10	7.9	1.23	0.5382
No	46								62.2	37	71.2	83	65.9		
Don’t know	22								29.7	11	21.2	33	26.2		
**Clinical knowledge**															
**Do you know that the most cases of toxoplasmosis in immunocompetent person are asymptomatic?**															
Yes	56								75.7	48	92.3	104	82.5	10.53	0.0052*
No	2								2.7	3	5.8	5	4.0		
Don’t know	16								21.6	1	1.9	17	13.5		
**Do you know that the seroconversion is:**															
Negative serology become positive during pregnancy*	50								67.6	44	84.6	94	74.6	5.58	0.0615
Positive serology become negative during pregnancy	7								9.5	4	7.7	11	8.7		
Don’t know	17								23.0	4	7.7	21	16.7		
**Do you know that the congenital toxoplasmosis defined when?**															
Passage of parasite from mother to fetus		45				60.8				31	59.6	76	60.3	0.76	0.6836
In case of seroconversion during pregnancy		22				29.7				18	34.6	40	31.7		
Don’t know		7				9.5				3	5.8	10	7.9		
**Do you know that the highest period of severity of lesion can be acquired in 1st trimester?**															
Yes			24				32.4			27	51.9	51	40.5	9.69	0.0079*
No			37				50.0			24	46.2	61	48.4		
Don’t know			13				17.6			1	1.9	14	11.1		
**Do you know that the highest-risk period of toxoplasmosis transmission can be acquired in the 3rd trimester?**															
Yes				12				16.2		6	11.5	18	14.3	5.38	0.0680
No				53				71.6		45	86.5	98	77.8		
Don’t know				9				12.2		1	1.9	10	7.9		
**Do you know that toxoplasmosis is an opportunist disease in immunocompromised persons?**															
Yes					19				25.7	15	28.8	34	27.0	0.99	0.6087
No					49				66.2	35	67.3	84	66.7		
Don’t know					6				8.1	2	3.8	8	6.3		

**Table 5:** prevention knowledge among doctors and nurses regarding toxoplasmosis

	Nurses		Doctors		Total N=126	%	X^2^	p
	N=74	(%)	N=52	(%)				
**Prevention knowledge**								
**Do you know that to avoid the transmission of toxoplasmosis in pregnant women they must:**								
**Washing hands when it’s necessary**								
Yes	68	91.9	52	100.0	120	95.2	4.42	0.1093
No	3	4.1	0	0.0	3	2.4		
Don’t know	3	4.1	0	0.0	3	2.4		
**Washing the fruit and vegetable product**								
Yes	69	93.2	52	100	121	96.0	3.66	0.1605
No	3	4.1	0	0.0	3	2.4		
Don’t know	2	2.7	0	0.0	2	1.6		
**No contact with cat**								
Yes	66	89.2	51	98.1	117	92.9	4.21	0.1218
No	3	4.1	1	1.9	4	3.2		
Don’t know	5	6.8	0	0.0	5	4.0		
**Eat the cooked meat**								
Yes	68	91.9	52	100	120	95.2	4.42	0.1093
No	1	1.4	0	0.0	1	0.8		
Don’t know	5	6.8	0	0.0	5	4.0		

## Discussion

Nowadays, researchers and global health practitioners use knowledge, attitude and practice surveys to gain insights necessary for health program design and implementation [14]. Medical health professionals as doctors and nurses play a significant role in promoting preventive behaviors to avoid acquiring toxoplasmosis during pregnancy. Therefore, the present study assessed the knowledge of two groups of MHPs, doctors and nurses regarding toxoplasmosis and is one of the few studies of this nature conducted in Morocco. Overall, most doctors and nurses had moderate knowledge of toxoplasmosis. However, less than half of respondents had low knowledge about toxoplasmosis. We observed a statistically significant difference between the nurses' level of knowledge and quality of work. The doctors had more knowledge than nurses, the majority of whom had low knowledge about toxoplasmosis. This finding is in agreement with that reported by AbdElmonaem *et al*., who reported that less than half of the participating nurses in Egypt had poor knowledge of toxoplasmosis [15]. In contrast, in Iraq, Khudair recorded that less than half of the nurses had moderate knowledge about toxoplasmosis and 20% of the participants had poor knowledge about toxoplasmosis [16]. The finding may be explained by the fact that doctors learn more about toxoplasmosis than nurses during their studies. Moreover, in our study, education was shown to be significantly associated with the quality of the profession. Indeed, MHPs, particularly nurses, need to be educated about toxoplasmosis. However, Angesom reported that most MHPs in Ethiopia did not receive training related to infectious diseases, especially toxoplasmosis leading to a lack of awareness and lack of attention given to the disease in health centers [17].

In the current survey, which involved doctors and nurses in Casablanca, nearly all participants agreed that the parasite *T. gondii* is the primary cause of toxoplasmosis and that cats are the sole host that *T. gondii* oocytes are excreted from (96.9% and 88%, respectively). According to a recent study conducted in Mexico, 89.6% and 83.8% of clinical laboratory experts correctly recognized *T. gondii* as the parasite that causes toxoplasmosis and cats as the specific hosts.

More than 80% of the participants accurately recognized risk factors such as ingestion of raw or undercooked meat, vegetable products and close contact with cats, according to the study's findings. While 65% were aware that untreated water is a source of toxoplasmosis. They also investigated the awareness among pregnant women and students in Casablanca and Rabat, Morocco, about untreated water being a risk factor and the route of transmission of toxoplasmosis [18,19]. Much research carried out all over the world has revealed the lack of knowledge among MHPs regarding untreated water as a risk factor and the mode of transmission. A few medical professionals in Nigeria and Ethiopia were aware that *T. gondii* infection is water-carried [20,21]. Additionally, a clinical laboratory professional in Mexico (13%) reported experiencing a similar finding [22].

Indeed, waterborne parasites have been linked to toxoplasmosis outbreaks in several studies conducted around the world [23,24]. According to Baldursson *et al*. (2011), toxoplasmosis outbreaks in Mexico were brought on by *T. gondii* oocysts in the water [23], and in a previous study from Brazil, waterborne *T. gondii* was implicated in an outbreak of the disease that affected 155 peoples and was caused by an underground tank reservoir delivering unfiltered water [24].

In the current study, only 8.3% of MPHs knew that the avidity test could be used to date the infection during the first months of pregnancy, even though the majority of MHPs (85.1%) correctly identified the detection of anti-*T. gondii* IgG and IgM antibodies as the standard test for the diagnosis of toxoplasmosis. Similar findings were reported previously in Mexico and the USA, where only 9.9% and 12.7% of health professionals, respectively, knew about the avidity test [25,26]. Hence, the avidity test does allow for the exclusion of evolutionary toxoplasmosis [27,28]. According to a study done in Rabat, Morocco, the use of the IgG avidity test allowed 83% of subjects with IgG and IgM positive serum for anti-*T. gondii* to be excluded from recent infection [12]. However, in cases where an amniocentesis test is positive, PCR is also used to diagnose *T. gondii* infection [26]. The use of PCR testing to confirm *T. gondii* infection or molecular tests to identify *T. gondii* DNA, however, are uncommon in Morocco, which explains the poor knowledge of MHPs about this diagnostic approach (27.8%) in the current study.

Additionally, nearly half of the surveyed doctors and nurses were aware that toxoplasmosis might be asymptomatic, indicating a worrisome lack of awareness and understanding among MHPs about the clinical manifestation of the infection. The majority of participating doctors and nurses, according to studies conducted in different nations, are aware that *T. gondii* infection is typically subclinical: 69.92% in Nigeria [21], 59.0% in Mexico [22], and 73.9% in Brazil [29]. Contrary to the current findings, de Moura *et al*. who examined healthcare professionals' perceptions of their knowledge of congenital toxoplasmosis in public healthcare facilities in Porto Alegre, reported that the majority of participants mentioned that the clinical manifestations of congenital toxoplasmosis included problems with the fetus and visual changes [30].

Less than half (44.7%) of participants knew that seroconversion toxoplasmosis in pregnant women can be treated to stop the parasite from getting to the fetus. In contrast, only 23% of respondents in Mexico were aware of the treatment for congenital toxoplasmosis, and 55.2% of clinicians there were unsure of what should be done in the event of seroconversion during pregnancy [26]. Many studies have documented a decrease in the severity of sequelae in those who received prenatal and postnatal care, even though the effectiveness of these treatments has not been conclusively proven [31].

Regarding the clinical knowledge about the risk periods during pregnancy, only (14.3%) of the respondents knew about the highest-risk period for the transmission of the parasite during pregnancy, whereas 40.5% knew about the highest-risk period of the gravity of the lesion for the fetus. Similarly, a low percentage of obstetricians and gynecologists in the USA and Mexico knew that the highest-risk period of congenital toxoplasmosis infection is the first trimester of pregnancy [22,32]. Indeed, vertical transmission of Toxoplasma infection to the fetus occurs during pregnancy or during delivery. The risk of the fetus developing major clinical signs decreases with increasing gestational age and the risk becomes higher than 80% in the final phase of the pregnancy [2].

## Conclusion

The present study is the first, to our knowledge, to report on the knowledge and attitudes of doctors and nurses in Casablanca about toxoplasmosis. They were found to have an average level of knowledge of toxoplasmosis. Therefore, education campaigns must be developed for MHPs to improve their awareness and knowledge about this disease.

### What is known about this topic

- The importance of toxoplasmosis disease in the world, which is an infection that can cause serious illness when contracted congenitally or when reactivated in immunocompromised individuals;
- The limited data about the knowledge and practice of toxoplasmosis among health professional in Morocco;
- The effectiveness of prenatal education program for the prevention of congenital toxoplasmosis.

### What this study adds

- Nurses and doctors have moderate knowledge of toxoplasmosis;
- Comparison of the two groups revealed that the nurses had less knowledge about toxoplasmosis than the doctors;
- The study highlights the need for awareness of knowledge on toxoplasmosis among health professionals in Casablanca.
